# Iron/folic acid supplementation during pregnancy prevents neonatal and under-five mortality in Pakistan: propensity score matched sample from two Pakistan Demographic and Health Surveys

**DOI:** 10.3402/gha.v9.29621

**Published:** 2016-02-11

**Authors:** Yasir B. Nisar, Michael J. Dibley

**Affiliations:** 1 United Nations Office for Project Services, Islamabad, Pakistan; 2 Sydney School of Public Health, University of Sydney, Sydney, Australia

**Keywords:** propensity score, antenatal care, iron/folic acid supplements, mortality, neonatal, under-five

## Abstract

**Background:**

Several epidemiological studies from low- and middle-income countries have reported a protective effect of maternal antenatal iron/folic acid (IFA) on childhood mortality.

**Objective:**

The current study aimed to evaluate the effect of maternal antenatal IFA supplementation on childhood mortality in Pakistan.

**Design:**

A propensity score–matched sample of 8,512 infants live-born within the 5 years prior to interview was selected from the pooled data of two Pakistan Demographic and Health Surveys (2006/07 and 2012/13). The primary outcomes were childhood mortality indicators and the main exposure variable was maternal antenatal IFA supplementation. Post-matched analyses used Cox proportional hazards regression and adjusted for 16 potential confounders.

**Results:**

Maternal antenatal IFA supplementation significantly reduced the adjusted risk of death on day 0 by 33% [adjusted hazard ratio (aHR)=0.67, 95% confidence interval (95% CI) 0.48–0.94], during the neonatal period by 29% (aHR=0.71, 95% CI 0.57–0.88), and for under-fives by 27% (aHR=0.73, 95% CI 0.60–0.89). When IFA was initiated in the first 4 months of pregnancy, the adjusted risk of neonatal and under-five deaths was significantly reduced by 35 and 33%, respectively. Twenty percent of under-five deaths were attributable to non-initiation of IFA in the first 4 months of pregnancy. With universal initiation of IFA in the first 4 months of pregnancy, 80,300 under-five deaths could be prevented annually in Pakistan.

**Conclusions:**

Maternal antenatal IFA supplementation significantly reduced neonatal and under-five deaths in Pakistan. Earlier initiation of supplements in pregnancy was associated with a greater prevention of neonatal and under-five deaths.

## Introduction

Every year, 6.3 million children die before reaching their fifth birthday worldwide (1). Of these deaths, 44% occur in the first 4 weeks of life or the neonatal period (1). The fourth Millennium Development Goal (MDG-4) aims to reduce under-five deaths by two-thirds by 2015, with a global target of 30 under-five deaths per 1,000 live births (1). Almost half of the under-five deaths occur in only five countries – India, Nigeria, Pakistan, Democratic Republic of the Congo, and China (1). The current under-five mortality rate in Pakistan is 89/1,000 live births (2), and the national MDG-4 target is 46/1,000 live births (1). During the last two decades in Pakistan (1990–2013), the under-five mortality rate has declined by 2.1% *per annum*, whereas the neonatal mortality rate has declined more slowly, at only 1.0% per year (1). The current rate of reduction of under-five mortality in Pakistan is insufficient to achieve the MDG-4 national targets (3), and substantial efforts will be required to achieve these targets.

The complications of preterm birth, infection, and birth asphyxia account for >90% of neonatal deaths in Pakistan (4). Maternal anaemia in the first or second trimester of pregnancy is associated with a substantially higher risk of low birthweight and preterm birth (5). Iron deficiency is the most common cause of anaemia during pregnancy and daily use of iron supplements during pregnancy significantly reduces the prevalence of maternal anaemia and risk of low birthweight (5, 6). The current World Health Organization (WHO) guidelines recommend provision of a daily prophylactic oral dose of iron (30–60 mg) and folic acid (400 µg) to all pregnant women, starting as early as possible during pregnancy (7). Iron/folic acid (IFA) supplementation during pregnancy has shown a preventive effect on neonatal and childhood mortality (8–16). However, the mechanism by which IFA supplementation reduces childhood mortality is not clear yet. Anaemia during pregnancy is associated with a higher risk of birth asphyxia (17, 18), which leads to neonatal mortality (19) and morbidity in the post-neonatal period (20). Both animal and human models have shown that anaemia is also associated with poor body temperature regulation (21), and hypothermia during the neonatal period is a major public health problem in high-altitude countries (22). Use of IFA supplements during pregnancy has been reported to significantly reduce maternal anaemia (5), low birthweight (5, 13, 23), and preterm delivery (23). Preterm delivery contributes to 14% of under-five deaths globally (19) and, compared to term births, preterm births lead to risk of neonatal and post-neonatal deaths that is 6.8 and 2.5 times higher, respectively (24). Secondary analysis of the 2006/07 Pakistan Demographic and Health Survey (PDHS) reported maternal perception of smaller-than-average birth size (proxy for low birthweight) as one of the determinants of neonatal mortality (25), and antenatal IFA supplementation significantly reduced the adjusted odds of smaller-than-average birth size and low birthweight (<2,000 g) in Pakistan (26). Thus, the reduction in risk of preterm birth, low birthweight, birth asphyxia, and poor temperature regulation are all potential mechanisms by which IFA supplementation during pregnancy can prevent under-five deaths. The current study aimed to examine the impact of IFA supplementation during pregnancy on neonatal, infant, and under-five mortality in Pakistan between 2002 and 2012.

## Methods

### Data sources

Pooled data from the 2006/07 (27) and 2012/13 PDHS (2) were used in the current study. The PDHS is a household cross-sectional survey that collects information from a nationally representative sample for a wide range of population, health, and nutrition indicators (28).

The sampling techniques used and the process of sample selection in these surveys have been described in detail elsewhere (2, 27, 28). To summarise, the PDHS used a multistage, stratified, cluster random sampling technique to collect demographic and health information by conducting interviews with ever-married women of reproductive age (15–49 years) and ever-married men. The PDHS was designed to produce estimates at the national, provincial, and urban/rural levels (2, 27). For each interviewed ever-married woman of reproductive age in both surveys, a complete birth history was recorded, and for the most recent birth during the last 5 years prior to the survey additional information about the woman's antenatal care (ANC), delivery, and postnatal care services were recorded. The birth history recorded all live births of a woman in chronological order, the date of the birth of each child, whether singleton or multiple births, the sex of the child, the survival status of the child on the day of the interview, and, if deceased, the date of death. The information collected in the PDHS was based on respondents’ recall. On average, the response rate was over 93% (2, 27).

**Fig. 1:**
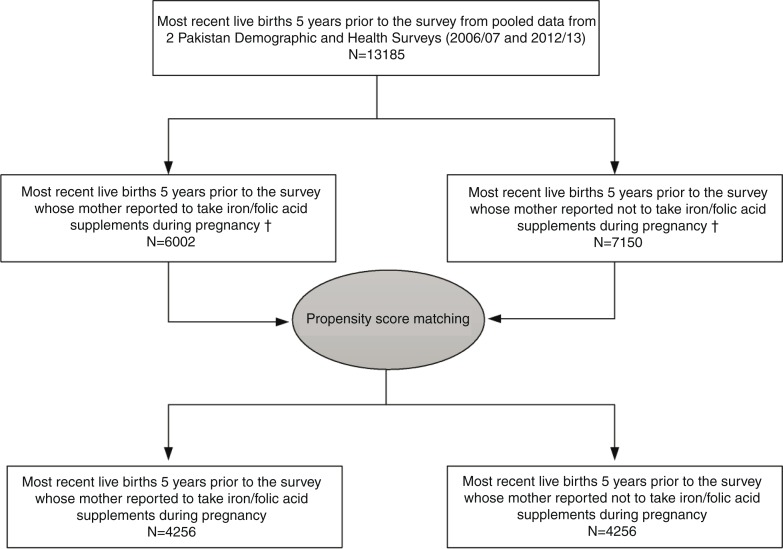
Process of selection of most recent live births in the 5 years prior to interview in two Demographic and Health Surveys based on propensity score matching in Pakistan, 2002–2012. †33 cases were excluded.

For the current analysis, we selected survival information from the 13,185 most recent live births in the 5 years prior to the interview date, consisting of 5,724 infants from the 2006/07 PDHS and 7,461 from the 2012/13 PDHS. We matched live-born infants in the 5 years prior to the interview date whose mothers used antenatal IFA supplements to those whose mothers did not use antenatal IFA supplements, based on their propensity score. The details of the selection of the sample for the current study are presented in Fig. 1.

### Propensity score matching

We created a propensity score using logistic regression to identify a set of predictor variables of maternal reported use of IFA supplements during pregnancy. The variables selected for evaluation were based on our previous study, which reported risk factors for IFA supplementation in Pakistan (29). We used eight variables to create the propensity score, as shown in Table 1. Thus, the propensity score was created from the probability of the mother's reported use of IFA supplements during pregnancy. We did not consider ANC visits, a predictor of IFA supplementation in Pakistan (29), in the set of covariates to create the propensity score because in our sample the household wealth index was strongly associated with ANC visits [odds ratio (OR)=3.06, 95% confidence interval (95% CI) 2.81–3.32, *p*<0.0001]. However, we created an alternate propensity score by replacing the household wealth index with the number of ANC visits. Similarly, we matched the mothers who used antenatal IFA supplements to those who did not use antenatal IFA supplements based on this alternate propensity score.

**Table 1:** Operational definition and categorisation of the covariates used for creating the propensity score and potential confounding factors

Variables	Definition and categorisation
Covariates used for propensity score development	
Place of residence	Respondent's place of residence (1: urban; 2: rural).
Pooled household wealth index	Composite index of household amenities using pooled PDHS data and a principal component analysis (35) of household assets. The wealth index was used to rank households across the two surveys into tertiles.
Maternal marital status	Marital status of the mother (1: currently married; 2: formerly married).
Maternal age at childbirth	Maternal age at childbirth (1: <20 years; 2: 20–24 years; 3: ≥25 years).
Maternal level of education attained	Maternal level of education attained (1: no education; 2: incomplete primary; 3: complete primary; 4: incomplete secondary; 5: complete secondary; 6: above secondary).
Maternal working status	Maternal employment status in the past 12 months prior to interview (1: not working; 2: working).
Birth status	Birth status of the child (1: singleton; 2: multiple).
Duration of recall	A continuous variable was constructed as difference in months between the date of the birth of the child and the date of interview.
Potential confounding factors	
Community-level and socio-economic status factors	
Province of residence	Province of the respondent (1: Punjab; 2: Sindh; 3: Khyber Pakhtunkhwa; 4: Balochistan).
Pooled household wealth index	Composite index of household amenities using pooled PDHS data and a principal component analysis (35) of household assets. The wealth index was used to rank households across the two surveys into tertiles.
Paternal level of education attained	Paternal level of education attained (1: no education; 2: incomplete/complete primary; 3: incomplete/complete secondary and above).
Paternal working status	Paternal employment status (1: not working; 2: working).
Average cluster coverage of BCG vaccine against tuberculosis	Average proportion of infants in the cluster who received BCG vaccination against tuberculosis.
Average cluster coverage of measles vaccination	Average proportion of infants in the cluster who received measles vaccination.
Maternal and newborn characteristics	
Maternal desire for pregnancy	Maternal intention to become pregnant (1: wanted later; 2: wanted no more; 3: wanted then).
Maternal perception of birth size	Subjective assessment of the respondent on the birth size (1: small; 2: smaller than average; 3: average; 4: larger than average; 5: large).
Birth rank and birth interval	Birth rank and birth interval of child (1: birth order 2 or 3, birth interval >2 years; 2: birth order 1; 3: birth order 2 or 3, birth interval ≤2 years; 4: birth order ≥4, birth interval >2 years; 5: birth order ≥4, birth interval ≤2 years).
Sex of child	Sex of the child (1: male; 2: female).
Timing of initiation of breastfeeding	Timing of initiation of breastfeeding (1: never breastfed; 2: <1 hour after birth; 3: 1–24 hours after birth; 4: >24 hours after birth).
Perinatal health services variables	
Number of antenatal care visits	Number of antenatal care visits (1: no antenatal care visits; 2: <4 antenatal care visits; 3: ≥4 antenatal care visits).
Place of delivery	Place of delivery (1: home; 2: health facility).
Delivery assistance	Birth attendance during delivery (1: traditional birth attendant/other untrained persons; 2: health professional).
Mode of delivery	Mode of delivery (1: non-caesarean section; 2: caesarean section).

BCG, Bacillus Calmette-Guerin vaccine; PDHS, Pakistan Demographic and Health Survey

We used the propensity score of a given mother who used IFA supplements during pregnancy, to match with the closest propensity score of a mother who did not use IFA supplements during pregnancy. This ‘nearest neighbour’ propensity score matching was employed without replacement. The calliper distance was the absolute difference in the propensity scores of matched mothers (30), and for a match it was required to be below a calliper width equal to 0.2 of the pooled standard deviation of the propensity score used to match the sample (31). Using this propensity score matching method, a sample of the 4,256 most recently live-born infants in the 5 years prior to interview whose mothers took IFA supplements during pregnancy were matched to a sample of infants whose mothers did not take IFA supplements during pregnancy (Fig. 1).

### Study factors

The mother's reported use of IFA supplements during the most recent pregnancy in the last 5 years prior to the interview date was the main exposure variable examined in the current study. The questions asked in the PDHS related to the use of IFA supplements were as follows: ‘During this pregnancy, were you given or did you buy any iron/folic acid tablets or iron syrup?’ and ‘During the whole pregnancy, for how many days did you take the tablets?’ A mother was classified as using IFA supplements during pregnancy if she reported taking IFA supplements for at least a day during pregnancy. However, in the current study we did not analyse the number of supplements taken during the pregnancy.

The effect of timing of the start of IFA supplementation (no IFA supplements; initiated in the first 4 months of pregnancy; or initiated after 4 months of pregnancy) on mortality outcomes was also assessed. We considered the time of the first ANC examination as a proxy for the start of IFA supplementation. We constructed a variable by combining ANC services and IFA supplementation (no IFA and no ANC; no IFA and any ANC services; or IFA with no or any ANC services). The question for the use of ANC services in the PDHS was ‘Did you see anyone for antenatal care for this pregnancy?’ The combination of use of any ANC services and IFA supplementation was considered to evaluate the effects of any IFA supplementation independent of the use of other ANC services.

### Primary and secondary outcomes

The primary study outcome was childhood mortality in four progressively longer cumulative time periods: *mortality at Day 0* defined as deaths on the first day of life, *neonatal mortality* defined as deaths after birth through 28 days of age, *infant mortality* defined as deaths after birth through 11 months of age, and *under-five mortality* defined as deaths after birth through 59 months of age (32).

A secondary outcome of the study was perception of birth size based on maternal recall. The question for the maternal perception of birth size in the PDHS was as follows: ‘When [NAME] was born, was he/she very large, larger than average, average, smaller than average, or small?’ The categories were pooled together to form a binary variable as ‘larger than or equal to the average birth size’ (including *large*, *larger than average*, and *average*) and ‘smaller than the average birth size’ (including *small* and *smaller than average*). Birthweight was recorded in only 12% of the sample and maternal perception of birth size was considered as a proxy for birthweight (33).

### Potential confounding factors and analytical framework

The potential confounding factors selected for the post-matched analysis were adapted from the Mosley and Chen framework of factors associated with child survival in developing countries (34). In total, we examined 16 potential confounding factors, which were classified into three main groups, community-level and socio-economic status variables, maternal and newborn characteristics, and perinatal healthcare service variables, as shown in Table 1. In addition, to adjust for secular trends in maternal iron status and development of perinatal healthcare services, a variable with the year of birth of the child was constructed and retained in all regression models.

The variables of birth rank and birth interval were combined as a five-category composite variable that consisted of first birth-rank infants, second or third birth-rank infants with a previous birth interval >2 years, second or third birth-rank infants with a previous birth interval ≤2 years, fourth or higher birth-rank infants with a previous birth interval >2 years, and fourth or higher birth-rank infants with a previous birth interval ≤2 years.

A household wealth index variable was constructed for household economic status by using the pooled PDHS data and a principal component analysis (35) of household assets and facilities, including type of toilet; main material of floor; source of drinking water; availability of electricity; possession of radio, television, fridge, or telephone; and bicycle. This household wealth index was used to rank households across the two surveys and was divided into tertiles for analysis.

### Ethical considerations

In each PDHS, informed consent was obtained from each respondent. The current secondary analysis protocol was approved by the Human Research Ethics Committee of the University of Sydney.

### Statistical analysis

Data analysis was conducted by using Stata 13.1 (StataCorp, College Station, TX, USA). Frequency tabulations were done for variables used to create the propensity score and potential confounding factors associated with the primary outcomes by mothers who reported taking IFA supplements and those who did not take IFA supplements during pregnancy. We used Cox proportional hazards regression to evaluate the associations between study factors and primary outcomes, initially with unadjusted regression analysis for each potential factor and later with adjusted analyses. We constructed multistage, multivariate Cox proportion regression models using a backward elimination technique to assess the independent effects of each of the factors after controlling for other covariates. In the first stage, community-level and socio-economic status variables were assessed for mortality outcomes, and non-significant variables (*p*>0.05) were removed. In the second stage, maternal and newborn characteristics were assessed in a model that already had significant community-level and socio-economic status variables for mortality outcomes. In the third stage, perinatal healthcare service variables were assessed in a model that contained significant community-level and socio-economic status variables and maternal and newborn characteristics associated with mortality outcomes. In the last stage, exposure variables (mother's reported use of IFA supplements during pregnancy, timing of initiation of IFA supplements during pregnancy) and combined variables (use of IFA supplements and use of any other ANC services) were assessed separately. The model used had significant community-level and socio-economic status variables, maternal and newborn characteristics, and perinatal health service variables. Hazard ratios (HRs) and their 95% CIs derived from adjusted Cox proportional hazards models were considered to examine the effect of the study factors on mortality. In statistical tests α≤0.05 was considered as significant, unless the variable had been *a priori* selected for inclusion, such as the year of birth. Plots of adjusted cumulative mortality for under-five mortality were generated based on the adjusted models.

We adjusted the propensity score post-matched analysis for sampling weights using additional models to evaluate the effect of IFA supplementation on mortality outcomes with ‘svy’ commands to adjust for the survey cluster sampling design. We used multistage multivariate Poisson regression analysis for the secondary outcome with a similar model-building technique to that described above for the primary outcome. Risk ratios and their 95% CIs derived from adjusted Poisson regression models were considered to examine the effect of the study factors on smaller-than-average birth size.

Population attributable risk (PAR) was calculated to assess total risk of mortality outcomes in the general population that was attributable to women who did not take IFA supplements during pregnancy and those who did not initiate supplements in the first 4 months of pregnancy. We assumed that the association between IFA supplementation and mortality was causal and that removal of IFA supplementation had no effect on the distribution of other risk factors for mortality. The following formula was used to calculate PAR (36–38):

PAR=Pe×[(aHR-1)/aHR]

where adjusted hazard ratio (aHR) was the adjusted hazard ratio for deaths of under-five children whose mother either did not take IFA supplements during pregnancy (this aHR is the inverse of the value reported for mothers who took IFA supplements) or did not initiate supplements in the first 4 months of pregnancy. Pe was the proportion of deaths of under-five children associated with having a mother who either did not take IFA supplements during pregnancy or who did not initiate supplements in the first 4 months of pregnancy. Based on estimates of PAR and the annual number of under-five deaths in Pakistan (1), we calculated the number of under-five deaths that could be prevented per year if all pregnant women in Pakistan took IFA supplements during pregnancy and if all pregnant women initiated supplements in the first 4 months of pregnancy.

## Results

Among the 8,512 most recent live-births in the 5 years prior to interview, there were 460 under-five deaths, 202 in mothers who took IFA supplements and 258 in mothers who did not take IFA supplements during pregnancy. Of the 460 under-five deaths, 336 were neonatal deaths. Among the 336 neonatal deaths, 103 deaths occurred on Day 0 and the remaining 233 deaths occurred between 1 and 28 days of life. Of the 4,256 women who used any antenatal IFA supplements, the median (interquartile range) number of days of antenatal IFA supplements use were 60 (30–120) days, and 532 (12.5%) consumed supplements for less than 15 days during their last pregnancy.

Table 2 presents the prevalence of covariates used to create the propensity score of the most recent live births in the 5 years prior to each interview in Pakistan 2002–2012, before and after the propensity score matching by the mother's reported use of IFA supplements during pregnancy. As expected, the prevalence of the covariates used to create the propensity score were similar after propensity score matching between infants whose mother took IFA supplements during pregnancy and those whose mother did not take IFA supplements.

**Table 2:** Prevalence of covariates used to create propensity score for the most recent live births in the 5 years prior to interview in Pakistan 2002–2012 by before and after propensity score matching and maternal antenatal iron/folic acid supplementation

	Pooled data from two PDHS		Propensity score matched sample	
		
	Maternal antenatal IFA supplementation		Maternal antenatal IFA supplementation	
		
Covariates	No *n* (%)	Yes *n* (%)	No *n* (%)	Yes *n* (%)
Place of residence				
Urban	2,272 (31.8)	2,990 (49.8)	1,708 (40.1)	1,657 (38.9)
Rural	4,878 (68.2)	3,012 (50.2)	2,548 (59.9)	2,599 (61.1)
Maternal age at childbirth				
<20 years	3,245 (45.4)	2,247 (37.4)	1,829 (43.0)	1,842 (43.3)
20–24 years	2,853 (39.9)	2,537 (42.3)	1,749 (41.1)	1,753 (41.2)
≥25 years	1,052 (14.7)	1,218 (20.3)	678 (15.9)	661 (15.5)
Maternal level of education attained				
No education	5,233 (73.2)	2,682 (44.7)	2,604 (61.2)	2,587 (60.8)
Incomplete primary	355 (5.0)	328 (5.5)	282 (6.6)	305 (7.2)
Complete primary	561 (7.8)	603 (10.0)	442 (10.4)	524 (12.3)
Incomplete secondary	408 (5.7)	579 (9.6)	363 (8.5)	403 (9.5)
Complete secondary	355 (5.0)	787 (13.1)	340 (8.0)	283 (6.7)
Above secondary	238 (3.3)	1,023 (17.0)	225 (5.3)	154 (3.6)
Maternal working status				
Not working	5,331 (74.6)	4,752 (79.2)	3,322 (78.1)	3,298 (77.5)
Working	1,818 (25.4)	1,247 (20.8)	934 (22.0)	958 (22.5)
Maternal marital status				
Currently married	7,050 (98.6)	5,959 (99.3)	4,221 (99.2)	4,218 (99.1)
Formerly married	100 (1.4)	43 (0.7)	35 (0.8)	38 (0.9)
Pooled household wealth index tertiles				
Highest	1,539 (21.5)	2,623 (43.7)	1,428 (33.6)	1,368 (32.1)
Middle	2,345 (32.8)	1,881 (31.3)	1,650 (38.8)	1,701 (40.0)
Lowest	2,994 (41.9)	1,202 (20.0)	1,178 (27.7)	1,187 (27.9)
Missing	272 (3.8)	296 (4.9)		
Birth status				
Singleton birth	7,083 (99.1)	5,918 (98.6)	4,201 (98.7)	4,202 (98.7)
Multiple births	67 (0.9)	84 (1.4)	55 (1.3)	54 (1.3)
Duration of recall (in months)^a^	22.8 (0.19)	21.5 (0.20)	21.9 (0.24)	22.6 (0.25)

IFA, iron/folic acid.

a Mean (SD).

Table 3 shows the distribution of the potential confounding factors for childhood mortality of the most recent live births 5 years prior to each interview by the mother's reported use of IFA supplements during pregnancy. Slightly over two-fifths of the fathers of infants whose mothers did not take IFA supplements during pregnancy and one-third of fathers of infants whose mothers took IFA supplements during pregnancy had no education. There was no use of ANC among 41% of the mothers who did not take IFA supplements, but only 10% of the mothers who took IFA supplements had no ANC. Thirty-eight percent of deliveries of mothers who did not take IFA supplements and 55% of the deliveries of mothers who took IFA supplements were facility-based.

**Table 3:** Prevalence of potential confounding factors for mortality outcomes of most recent live births in the 5 years prior to interview in Pakistan 2002–2012 by maternal antenatal iron/folic acid supplementation

	Maternal antenatal IFA supplementation	
	
Variables	No *n* (%)	Yes *n* (%)
Community-level and socio-economic status variables		
Province of residence		
Punjab	1,932 (45.4)	1,592 (37.4)
Sindh	908 (21.3)	1,189 (27.9)
Khyber Pakhtunkhwa	719 (16.9)	1,083 (25.5)
Balochistan	697 (16.4)	392 (9.2)
Paternal level of attained education		
No education	1,755 (41.2)	1,430 (33.6)
Incomplete/complete primary	1,130 (26.6)	1,170 (27.5)
Incomplete/complete secondary or above	1,368 (32.1)	1,652 (38.8)
Missing	3 (0.1)	4 (0.1)
Paternal working status		
Not working	145 (3.4)	137 (3.2)
Working	4,109 (96.6)	4,118 (96.8)
Missing	2 (0.1)	1 (0.0)
Average cluster coverage of BCG vaccination against tuberculosis^a^	0.75 (0.004)	0.80 (0.003)
Average cluster coverage of measles vaccination^a^	0.46 (0.004)	0.50 (0.004)
Maternal and newborn characteristics		
Maternal desire for pregnancy		
Wanted later	439 (10.3)	476 (11.2)
Wanted no more	513 (12.1)	512 (12.0)
Wanted then	3,298 (77.5)	3,264 (76.7)
Missing	6 (0.1)	4 (0.1)
Sex of the child		
Male	2,221 (52.2)	2,267 (53.3)
Female	2,035 (47.8)	1,989 (46.7)
Birth rank and birth interval		
Birth order 2 or 3, >2-year interval	892 (21.0)	931 (21.9)
Birth order 1	643 (15.1)	798 (18.8)
Birth order 2 or 3, ≤2-year interval	548 (12.9)	534 (12.6)
Birth order ≥4, >2-year interval	1,498 (35.2)	1,456 (34.2)
Birth order ≥4, ≤2-year interval	675 (15.9)	537 (12.6)
Maternal perception of birth size		
Small	372 (8.7)	348 (8.2)
Smaller than average	769 (18.1)	730 (17.2)
Average	2,488 (58.5)	2,521 (59.2)
Larger than average	532 (12.5)	549 (12.9)
Large	79 (1.9)	104 (2.4)
Missing	16 (0.4)	4 (0.1)
Timing of initiation of breastfeeding		
Never breastfed	224 (5.3)	218 (5.1)
<1 hour after birth	1,834 (43.1)	1,771 (41.6)
1–24 hours after birth	1,162 (27.3)	1,212 (28.5)
>24 hours after birth	1,029 (24.2)	1,050 (24.7)
Missing	7 (0.2)	5 (0.1)
Perinatal health services variables		
Number of ANC visits		
No ANC visits	1,742 (40.9)	411 (9.7)
1–3 ANC visits	1,579 (37.1)	1,904 (44.7)
≥4 ANC visits	920 (21.6)	1,900 (44.6)
Missing	15 (0.4)	41 (1.0)
Place of delivery		
Home	2,658 (62.5)	1,897 (44.6)
Health facility	1,598 (37.5)	2,359 (55.4)
Delivery assistance		
Traditional birth attendant/other untrained person	2,421 (56.9)	1,773 (41.7)
Health professionals	1,831 (43.0)	2,477 (58.2)
Missing	4 (0.1)	6 (0.1)
Mode of delivery		
Non-caesarean section	3,916 (92.0)	3,726 (87.6)
Caesarean section	336 (7.9)	524 (12.3)
Missing	4 (0.1)	6 (0.1)

ANC, antenatal care.

a Mean (SD).

The risk factors for under-five mortality of the most recent live births in the 5 years prior to each interview are shown in Table 4. Infants who were living in the province of Sindh (aHR=1.37, 95% CI 1.09–1.74), who belonged to the lowest household wealth index tertile (aHR=1.86, 95% CI 1.41–2.44), whose mother wanted no more children (aHR=1.53, 95% CI 1.05–2.21), female infants (aHR=1.23, 95% CI 1.02–1.49), and who were at least fourth in birth rank with ≤2 years of birth interval (aHR=1.65, 95% CI 1.19–2.27) had significantly higher risk of under-five mortality than their counterparts without these risk factors. Infants who were breastfed compared to never breastfed and whose mother had four or more ANC visits compared to no ANC visits had significantly lower risk of under-five mortality.

**Table 4:** Risk factors associated with under-five mortality of most recent live births 5 years prior to interview in Pakistan 2002–2012: results of multivariate Cox proportional hazard regression analyses

			Adjusted^a^	
			
Variables	Number of live births	Number of under-five deaths	HR (95% CI)	*p*
Province of residence				0.001
Punjab	3,524	183	1.00 (reference)	
Sindh	2,097	137	1.37 (1.09–1.74)	
Khyber Pakhtunkhwa	1,802	75	0.76 (0.57–1.02)	
Balochistan	1,089	65	1.03 (0.75–1.42)	
Pooled household wealth index tertiles				<0.0001
Highest	2,796	116	1.00 (reference)	
Middle	3,351	168	1.32 (1.02–1.71)	
Lowest	2,365	176	1.86 (1.41–2.44)	
Maternal desire for pregnancy				0.024
Wanted later	915	36	1.00 (reference)	
Wanted no more	1,025	44	1.53 (1.05–2.21)	
Wanted then	6,562	380	1.11 (0.69–1.78)	
Sex of the child				0.032
Male	4,488	229	1.00 (reference)	
Female	4,024	231	1.23 (1.02–1.49)	
Birth rank and birth interval				0.016
Birth order 2 or 3, >2-year interval	1,823	78	1.00 (reference)	
Birth order 1	1,441	86	1.24 (0.90–1.70)	
Birth order 2 or 3, ≤2-year interval	1,082	66	1.57 (1.12–2.21)	
Birth order ≥4, >2-year interval	2,954	138	1.21 (0.90–1.62)	
Birth order ≥4, ≤2-year interval	1,212	92	1.65 (1.19–2.27)	
Timing of initiation of breastfeeding				<0.0001
Never breastfed	442	217	1.00 (reference)	
<1 hour after birth	3,605	102	0.04 (0.03–0.05)	
1–24 hours after birth	2,374	77	0.04 (0.03–0.05)	
>24 hours after birth	2,079	63	0.04 (0.03–0.05)	
Number of antenatal care visits				0.014
No ANC visits	2,153	141	1.00 (reference)	
1–3 ANC visits	3,483	183	0.82 (0.65–1.04)	
≥4 ANC visits	2,820	135	0.66 (0.51–0.87)	

76 missing values were excluded from the analysis. CI, confidence interval; HR, hazard ratio.

a Adjusted for province of residence, pooled household wealth index, average coverage of measles vaccination, paternal level of education attained, paternal working status, maternal desire for pregnancy, sex of the child, birth rank and birth interval, maternal perception of birth size, timing of initiation of breastfeeding, number of antenatal care visits, place of delivery, delivery assistance, mode of delivery, and year of birth.

**Fig. 2:**
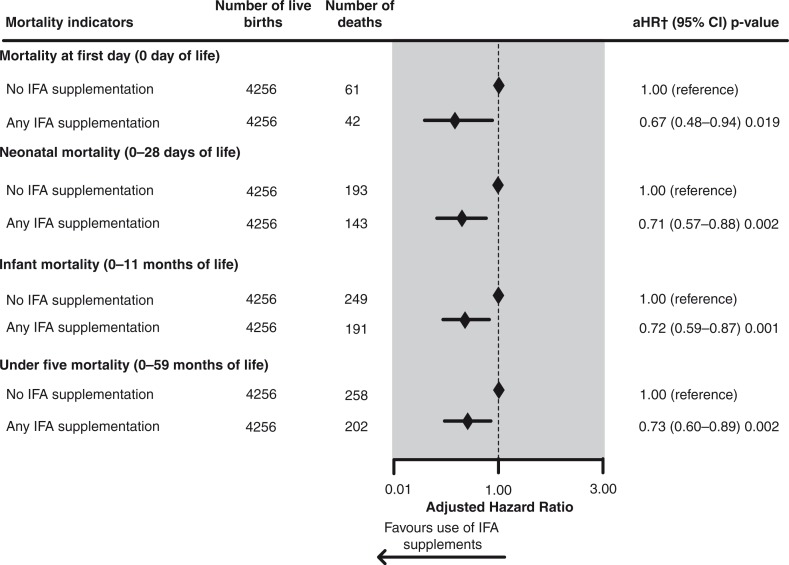
Effect of maternal antenatal iron/folic acid supplementation on childhood mortality indicators of most recent live births in the 5 years prior to interview in Pakistan, 2002–2012: results of multivariate Cox proportional hazards regression analyses. Seventy-six missing values were excluded from the analysis. †Adjusted for province of residence, pooled household wealth index, average coverage of BCG vaccination against tuberculosis (for mortality in neonatal and infant period), average coverage of measles vaccination (for under-five mortality), paternal level of education attained, paternal working status, maternal desire for pregnancy, sex of the child, birth rank and birth interval, maternal perception of birth size, timing of initiation of breastfeeding, number of antenatal care visits, place of delivery, delivery assistance, mode of delivery, and year of birth.

The effect of mother's reported use of IFA supplements during pregnancy on childhood mortality indicators of the most recent live births in the 5 years prior to each interview in Pakistan 2002–2012, is shown in Fig. 2. In infants whose mother took IFA supplements during pregnancy the adjusted risk of mortality at Day 0 of life was significantly reduced by 33% (aHR=0.67, 95% CI 0.48–0.94), compared to those whose mother did not take IFA supplements during pregnancy. The adjusted risk of neonatal mortality was significantly reduced by 29% (aHR=0.71, 95% CI 0.57–0.88) in infants whose mother took IFA supplements during pregnancy compared to those whose mother did not take IFA supplements. Similarly, the adjusted risk of infant death and under-five death were significantly reduced by 28% (aHR=0.72, 95% CI 0.59–0.87) and 27% (aHR=0.73, 95% CI 0.60–0.89), respectively, in infants whose mother took IFA supplements, compared to those whose mother did not take IFA supplements during pregnancy.

When mortality models were adjusted for potential confounders as well as for sampling design used in the survey (weighted analysis), the maternal IFA supplementation during pregnancy showed a slightly lower, but significant, extent of mortality-preventing effects on all mortality indicators examined in the current study (Supplementary Fig. 1).

The prevalence of covariates used to create the alternate propensity score of the most recent live births in the 5 years prior to each interview were similar after propensity score matching between infants whose mother took IFA supplements during pregnancy and those whose mother did not take IFA supplements (Supplementary Table 1). A total of 3,879 live-born infants in the 5 years prior to the interview date whose mothers used IFA supplements had matched mothers who did not use IFA supplements. As expected, the prevalence of the covariates used to create the alternate propensity score was similar. With maternal IFA supplementation during pregnancy, the adjusted risk of mortality at Day 0 was significantly reduced by 31% (aHR=0.69, 95% CI 0.48–0.98).We found borderline significant reductions, by 21% (aHR=0.79, 95% CI 0.62–1.01) and by 19% (aHR=0.81, 95% CI 0.66–1.00), in neonatal and infant mortality, respectively. Maternal IFA supplementation during pregnancy significantly reduced the adjusted risk of under-five mortality, by 21% (aHR=0.80, 95% CI 0.65–0.98) (Supplementary Table 2).

**Fig. 3:**
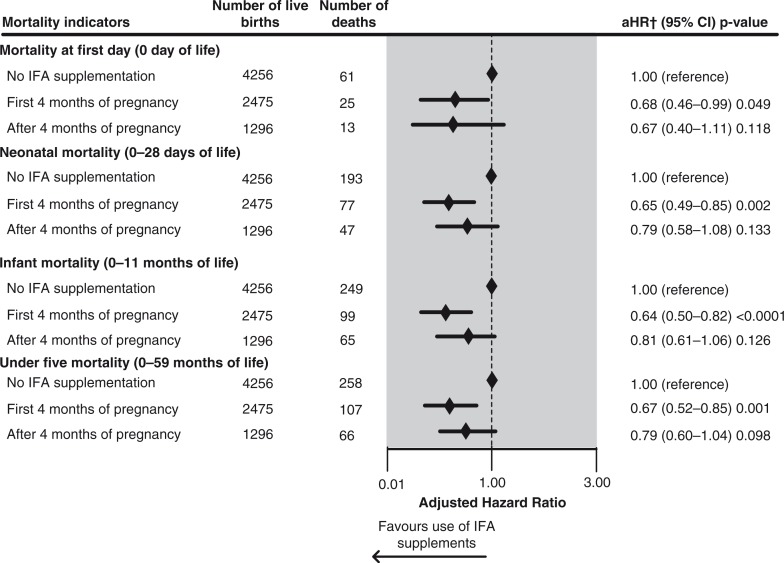
Effect of timing of initiation of maternal antenatal iron/folic acid supplementation on childhood mortality indicators of most recent live births in the 5 years prior to interview in Pakistan 2002–2012: results of multivariate Cox proportional hazards regression analyses. Five hundred twenty-one missing values were excluded from the analysis. †Adjusted for province, pooled household wealth index, average coverage of BCG vaccination against tuberculosis (for mortality in neonatal and infant period), average coverage of measles vaccination (for under-five mortality), paternal level of education attained, paternal working status, maternal desire for pregnancy, sex of the child, birth rank and birth interval, maternal perception of birth size, timing of initiation of breastfeeding, number of antenatal care visits, place of delivery, delivery assistance, mode of delivery, and year of birth.

Figure 3 presents the effect of timing of initiation of IFA supplements during pregnancy on childhood mortality indicators. When IFA supplements were started in the first 4 months of pregnancy, the adjusted risk of mortality at Day 0 was significantly reduced by 32% (aHR=0.68, 95% CI 0.46–0.99). Further, the adjusted risk of neonatal mortality was significantly reduced by 35% (aHR=0.65, 95% CI 0.49–0.85) and infant mortality by 36% (aHR=0.64, 95% CI 0.50–0.82) with initiation of IFA in the first 4 months of pregnancy. The adjusted risk of under-five mortality was significantly reduced by 33% (aHR=0.67, 95% CI 0.52–0.85) when supplements were started in the first 4 months of pregnancy.

**Fig. 4:**
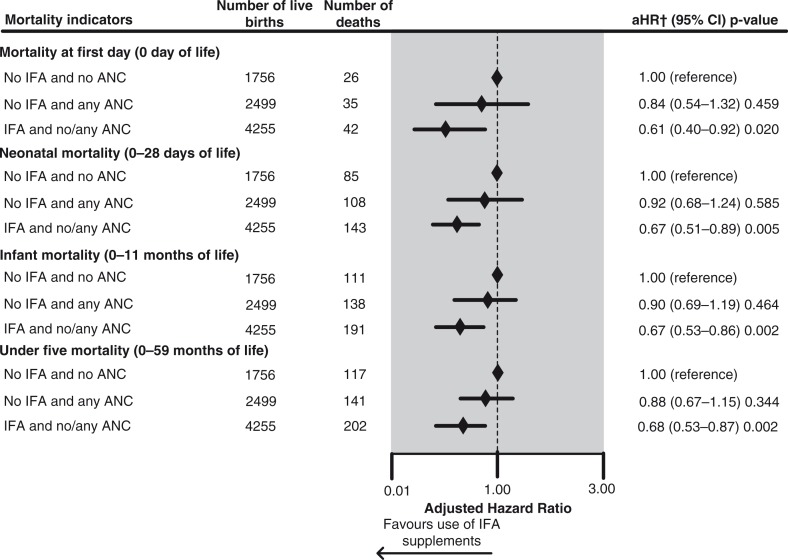
Effect of combination of maternal antenatal iron/folic acid supplementation and other antenatal care services used on childhood mortality indicators of most recent live births in the 5 years prior to interview in Pakistan, 2002–2012: results of multivariate Cox proportional hazards regression analyses. Seventy-six missing values were excluded from the analysis. †Adjusted for province of residence, pooled household wealth index, average coverage of BCG vaccination against tuberculosis (for mortality in neonatal and infant period), average coverage of measles vaccination (for under-five mortality), paternal level of education attained, paternal working status, maternal desire for pregnancy, sex of the child, birth rank and birth interval, maternal perception of birth size, timing of initiation of breastfeeding, number of antenatal care visits, place of delivery, delivery assistance, mode of delivery, and year of birth.

The effect of the combination of IFA supplements and use of any other ANC services on childhood mortality indicators is shown in Fig. 4. Pakistani infants whose mothers used IFA supplements, with or without the use of any other ANC services, had an adjusted risk of mortality at Day 0 that was significantly reduced by 39% (aHR=0.61, 95% CI 0.40–0.92). Further, the adjusted risk of neonatal mortality was significantly reduced by 33% (aHR=0.67, 95% CI 0.51–0.89), infant mortality by 43% (aHR=0.57, 95% CI 0.53–0.86), and under-five mortality by 32% (aHR=0.68, 95% CI 0.53–0.87) in infants whose mothers used IFA supplements, with or without the use of any other ANC services, compared to those whose mothers used neither IFA nor any other ANC services. Further, use of any other ANC services without using IFA supplements had no significant effect on the adjusted risk of the four mortality indicators.

**Fig. 5:**
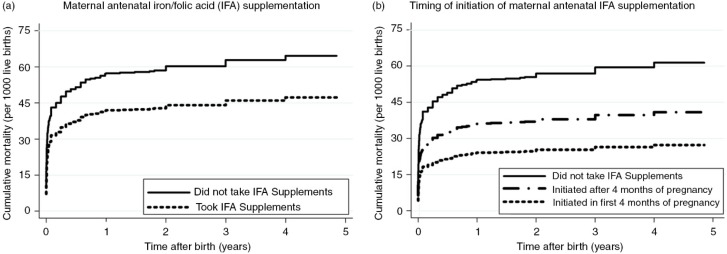
Plot of adjusted cumulative mortality for under-five children by (a) maternal antenatal iron/folic acid supplementation; (b) timing of initiation of maternal antenatal iron/folic acid supplementation. Results of the Cox proportional hazards regression analysis were adjusted for province, pooled household wealth index, average coverage of BCG vaccination against tuberculosis (for mortality in neonatal and infant period), average coverage of measles vaccination (for under-five mortality), paternal level of education attained, paternal working status, maternal desire for pregnancy, sex of the child, birth rank and birth interval, maternal perception of birth size, timing of initiation of breastfeeding, number of antenatal care visits, place of delivery, delivery assistance, mode of delivery, and year of birth.

Plots of the adjusted cumulative mortality for children under-five by mother's use of IFA supplements during pregnancy and timing of initiation of IFA supplements are shown in Fig. 5a and b, respectively. The plot was based on the findings of the model reported in Fig. 2 for mother's reported use of IFA supplements during pregnancy and in Fig. 3 for the timing of initiation of IFA supplementation during pregnancy. The cumulative mortality in children under-five was substantially lower in infants whose mothers reported taking IFA supplements during pregnancy or those who initiated the supplements in the first 4 months of pregnancy. The difference in cumulative mortality was evident from birth and progressively increased throughout the under-five period.

The PAR estimates found that in Pakistan 15% (PAR=0.15, 95% CI 0.04–0.26) of under-five deaths were attributed to non-use of IFA supplements during pregnancy and 20% (PAR=0.20, 95% CI 0.04–0.35) of under-five deaths to not initiating the supplements in the first 4 months of pregnancy. Based on the estimated PAR, 58,500 (95% CI 14,000–104,000) under-five deaths *per annum* could be prevented in Pakistan if all pregnant women took IFA supplements during pregnancy and 80,300 (95% CI 16,800–139,000) if all women started the supplements in the first 4 months of their pregnancies.

With maternal antenatal IFA supplementation, the risk of smaller-than-average birth size infants was significantly reduced by 9% (aRR=0.91, 95% CI 0.85–0.98) compared to no supplementation after adjusting for potential confounding factors. The adjusted risk of smaller-than-average birth size infants was significantly reduced by 17% (aRR=0.83, 95% CI 0.76–0.91) when the supplements were initiated in the first 4 months of pregnancy. The adjusted risk of smaller-than-average birth size infants was significantly reduced by 11% (aRR=0.89, 95% CI 0.81–0.97) with maternal antenatal IFA supplementation with or without use of any other ANC services in Pakistan. We found a non-significant effect on the adjusted risk of smaller-than-average birth size when any other ANC services were used without using IFA supplements (Supplementary Fig. 2).

## Discussion

### Main findings and their significance

The current study found that in Pakistan between 2002 and 2012 the adjusted risk of childhood mortality indicators for all four progressively longer cumulative time periods was significantly reduced in children whose mothers reported taking IFA supplements during pregnancy. When the supplements were started during the first 4 months of pregnancy, there were greater reductions in the adjusted risk of all the mortality indicators examined. There was no significant effect of maternal antenatal IFA supplementation on any mortality indicators when a mother started the supplements after 4 months of pregnancy. Further, we found that there were no significant effects on mortality with mothers who used ANC services without antenatal IFA supplementation. We also created an alternate propensity score by considering ANC visits, and our findings showed that maternal IFA supplementation during pregnancy had a significant or borderline significant protective effect on all mortality indicators examined in the study. It is argued that the protective effect of IFA supplementation on mortality could be due to ANC visits (39). However, the findings of our alternate propensity score matched sample showed that IFA had an independent effect on mortality irrespective of the number of ANC visits. Further, we found a significant adjusted risk reduction in smaller-than-average birth size infants with maternal antenatal IFA supplementation. The adjusted risk reduction for smaller-than-average birth size infants was only significant when the supplements were initiated in the first 4 months of pregnancy. Further, there was no significant effect on smaller-than-average birth size infants with mothers who only used ANC services without antenatal IFA supplementation.

The current study is the first analysis to report the effect of maternal antenatal IFA supplementation on under-five mortality in Pakistan using propensity score matched samples from the pooled data of two PDHS. According to the current WHO guidelines, all pregnant women should be given IFA supplements and start as early as possible during pregnancy (7). However, during the last decade (2002–2012) the coverage of antenatal IFA supplementation has remained static and low (approximately 45%) in Pakistan (2, 27). The current findings provide key evidence to policymakers and programme mangers working in maternal, newborn, and child health of the need to improve the current coverage of antenatal IFA supplementation in Pakistan to improve progress toward achieving the MDG-4 national targets.

### Strengths and limitations

The main strength of the study was the use of a propensity score matched sample analysis, which mimics some of the characteristics of a randomised controlled trial (RCT) (30). An appropriate set of covariates was used to create the propensity score; they were selected primarily based on an earlier analysis of factors associated with IFA supplementation in Pakistan (29). To minimise recall bias, a variable recording the duration of recall from the birth until the interview was also used to develop the propensity score. The sample was matched using a calliper of width equal to 0.2 to the pooled standard deviation of the logit of the propensity score, which eliminates approximately 99% of the bias due to the measured confounders (30). For the post-matched analysis, we used Cox proportional hazard models to examine the effect of maternal antenatal IFA supplementation on mortality, adjusted for several potential founders. Use of ANC services has previously been reported as a predictor for IFA supplementation (29). We did not consider it in the set of covariates to create the propensity score. We adjusted all the propensity score post-matched analyses for the number of ANC visits, as suggested by others (39). We also assessed the independent effect of IFA supplementation on mortality indictors with or without any other ANC services used, by creating a composite variable. Further, we created an alternate propensity score considering the number of ANC visits and matched the sample based on alternate propensity score matching. To minimise recall bias, we restricted analyses to the most recent live-births within the 5 years prior to the interview date (32, 40, 41), as there was no difference in risk factors associated with antenatal IFA supplementation if analysis was restricted to either the last 5 years or the last 3 years (29). Both PDHS were conducted by the same organisation using the same core questionnaire to collect data with good quality control measures (2, 27). In the two PDHS (2, 27) used there were only slight variations in the neonatal, infant, and under-five mortality rates observed for the same reference period of time in each survey, suggesting good quality data collection for births and deaths.

The information gathered in the PDHS about child births, deaths, and maternal antenatal IFA supplementation were based on maternal recall and could not be validated, which is the major limitation of the current study. The recorded information of child births and deaths, however, are core measurements of DHS and the methods used have been carefully examined over two decades (42). There is a possibility of underreporting of child deaths, as birth histories and child survival information were only collected from surviving mothers and there is a strong association between maternal and child deaths (32). Anthropometric status of children, a potential predictor of child mortality, could not be examined in the analyses because anthropometric measurements were not available for all children. Further, any improvement in maternal iron status could not be assessed from the DHS data. With our model-building approach, there was a chance of missing interactions among some explanatory variables entered and eliminated at different stages of the model building. Nonetheless, use of this hierarchical modelling approach has been recommended for such analyses (43). Finally, PAR estimates depend on the prevalence of exposure, which might vary across populations.

### Comparison with other studies

The current study findings are in agreement with our earlier analysis using traditional multivariate Cox proportional hazards regression from Pakistan (15), Nepal (15), and Indonesia (14). Secondary analyses of data from two DHS from Pakistan (2006/07 and 2012/13) and two from Nepal (2006 and 2011) reported a significant reduction of 23 and 51%, respectively, in the adjusted risk of early neonatal mortality with maternal antenatal IFA supplementation. Further, the mortality prevention effect in both countries was greatest with earlier initiation of supplements and a greater number of supplements used during pregnancy (15). Previously, pooled data from four Indonesian DHS (1994, 1997, 2002–03, and 2007) found a significant reduction by 60, 39, and 34% in the risk of mortality on the first day of life, neonatal, and under-five mortality, respectively, with maternal antenatal IFA supplementation. When the supplements were started in the first trimester of pregnancy, the adjusted risk of under-five mortality was significantly reduced by 38% (14). A cluster RCT in China found a significant reduction by 54% in the risk of early neonatal deaths and by 47% in the risk of neonatal deaths in infants whose mothers took IFA supplements during pregnancy compared to those whose mothers took folic acid alone (23). A cluster RCT of four different combinations of micronutrient supplements in pregnancy in Nepal found a significant reduction by 47% in the risk of death for infants aged 0–3 months among preterm births whose mothers took IFA supplements during pregnancy (13). The follow-up study of the trial found a significant reduction by 31% in the risk of mortality between birth and 7 years of age in children whose mother consumed IFA supplements during their pregnancy (12).

### Impact of IFA supplementation on child survival

We found that 15% of under-five deaths were attributed to non-use of antenatal IFA supplements and 20% were due to non-initiation of supplements in the first 4 months of pregnancy. Secondary analyses of DHS from Indonesia found that 14% (14) of under-five deaths were attributable to non-use of antenatal IFA supplements. We estimated each year 58,500 under-five deaths could be averted with universal maternal antenatal IFA supplementation and 80,300 under-five deaths could be prevented with universal initiation of IFA in the first 4 months of pregnancy in Pakistan. Similarly, we found a greater effect on the adjusted risk reduction of smaller-than-average birth size infants when the supplements were initiated in the first 4 months of pregnancy. Pakistan, like many low- and middle-income countries, has for a long time had a policy of universal, daily antenatal IFA supplementation starting at the beginning of the second trimester of pregnancy and continuing throughout pregnancy. However, Pakistan has the lowest coverage of antenatal IFA supplementation in South Asia (44–46), and there has been hardly any change in the prevalence of coverage of antenatal IFA supplementation during the last decade (2, 27). Hence, there is a need to strengthen the IFA supplementation programmes in Pakistan. Several countries have improved their national IFA supplementation programme and subsequently increased the coverage of antenatal IFA supplementation through distribution of IFA by community health worker programmes (47–49). Pakistan has one of the largest community health worker programmes (the Lady Health Workers Program), and female health workers in their programme area are responsible to provide IFA supplements to pregnant women as part of ANC services in resource-poor communities (50). However, there is a need to strengthen the logistic system of IFA supplementation (51), for retraining to improve knowledge and counselling skills of female health workers about IFA supplementation (51), and for increasing community awareness (51).

To conclude, the current study found that infants in Pakistan whose mothers took antenatal IFA supplements had a significantly lower adjusted risk of neonatal, infant, and under-five death. Although we used a propensity score matched sample to reduce bias, there remains a need to conduct large-scale community-based RCTs to evaluate the effectiveness of maternal antenatal IFA supplementation on childhood mortality in Pakistan.

## Supplementary Material

Iron/folic acid supplementation during pregnancy prevents neonatal and under-five mortality in Pakistan: propensity score matched sample from two Pakistan Demographic and Health Surveys
