# Supplementary material for: Iron/folic acid supplementation during pregnancy prevents neonatal and under-five mortality in Pakistan: propensity score matched sample from two Pakistan Demographic and Health Surveys
Source: Glob Health Action. 2016 Feb 11;9:10.3402/gha.v9.29621. doi: 10.3402/gha.v9.29621 (PMC4752592; doi:10.3402/gha.v9.29621)
Supplement: Iron/folic acid supplementation during pregnancy prevents neonatal and under-five mortality in Pakistan: propensity score matched sample from two Pakistan Demographic and Health Surveys [file GHA-9-29621-s001.docx]

# Supplementary material

**Table S1: Prevalence of Covariates used to create Propensity Score for the Most Recent Live Births 5 Years prior to Interview in Pakistan 2002–2012 by Before and After Alternate Propensity Score Matching and Maternal Antenatal Iron/folic Acid Supplementation**

| **Covariates** | **Pooled data from two PDHS** | | **Alternate propensity score matched sample** | |
| --- | --- | --- | --- | --- |
|  | **Maternal antenatal IFA supplementation** | | **Maternal antenatal IFA supplementation** | |
|  | **No**  **n (%)** | **Yes**  **n (%)** | **No**  **n (%)** | **Yes**  **n (%)** |
| **Place of residence** |  |  |  |  |
| Urban | 2272 (31.8) | 2990 (49.8) | 1543 (39.8) | 1476 (38.1) |
| Rural | 4878 (68.2) | 3012 (50.2) | 2336 (60.2) | 2403 (61.9) |
| **Maternal age at childbirth** |  |  |  |  |
| <20 years | 3245 (45.4) | 2247 (37.4) | 1666 (42.9) | 1741 (44.9) |
| 20 – 24 years | 2853 (39.9) | 2537 (42.3) | 1592 (41.1) | 1553 (40.0) |
| ≥25 years | 1052 (14.7) | 1218 (20.3) | 621 (16.0) | 585 (15.1) |
| **Maternal level of attained education** |  |  |  |  |
| No education | 5233 (73.2) | 2682 (44.7) | 2322 (59.9) | 2408 (62.1) |
| Incomplete primary | 355 (5.0) | 328 (5.5) | 232 (6.0) | 244 (6.3) |
| Complete primary | 561 (7.8) | 603 (10.0) | 434 (11.2) | 388 (10.0) |
| Incomplete secondary | 408 (5.7) | 579 (9.6) | 322 (8.3) | 336 (8.7) |
| Complete secondary | 355 (5.0) | 787 (13.1) | 331 (8.5) | 331 (8.5) |
| Above secondary | 238 (3.3) | 1023 (17.0) | 238 (6.1) | 172 (4.4) |
| **Maternal working status** |  |  |  |  |
| Not working | 5331 (74.6) | 4752 (79.2) | 3034 (78.2) | 2943 (75.9) |
| Working | 1818 (25.4) | 1247 (20.8) | 845 (21.8) | 936 (24.1) |
| **Maternal marital status** |  |  |  |  |
| Currently married | 7050 (98.6) | 5959 (99.3) | 3843 (99.1) | 3842 (99.1) |
| Formerly married | 100 (1.4) | 43 (0.7) | 36 (0.9) | 37 (0.9) |
| **Number of antenatal care visits** |  |  |  |  |
| No or less than 4 visits | 5944 (83.1) | 2785 (46.4) | 2674 (68.9) | 2744 (70.7) |
| 4 or more visits | 1205 (16.9) | 3216 (53.6) | 1205 (31.1) | 1135 (29.3) |
| **Birth status** |  |  |  |  |
| Singleton birth | 7083 (99.1) | 5918 (98.6) | 3836 (98.9) | 3833 (98.8) |
| Multiple births | 67 (0.9) | 84 (1.4) | 43 (1.1) | 46 (1.2) |
| **Duration of recall (in months)**† | 22.8 (0.19) | 21.5 (0.20) | 21.1 (0.24) | 22.3 (0.26) |

†Mean (SD)

IFA: iron/folic acid; PDHS: Pakistan Demographic and Health Survey.

**Table S2: Effect of Maternal Antenatal Iron-Folic Acid Supplementation on Childhood Mortality Indicators of Most Recent Live Births 5 Years prior to Interview in Pakistan 2002–2012 using the Alternate Propensity Score Matched Sample: Results of Multivariate Cox Proportional Hazard Regression Analyses**

|  | **Number of live births** | **Number of deaths** | **Adjusted^†^** | |
| --- | --- | --- | --- | --- |
| **Mortality indicator** |  |  | **HR (95% CI)** | ***P*** |
| **Mortality at first day (0 day of life)** |  |  |  |  |
| No IFA supplementation | 3879 | 57 | 1.00 (reference) |  |
| Any IFA supplementation | 3879 | 38 | 0.68 (0.48–0.98) | 0.040 |
| **Neonatal mortality (0-28 day of life)** |  |  |  |  |
| No IFA supplementation | 3879 | 136 | 1.00 (reference) |  |
| Any IFA supplementation | 3879 | 95 | 0.79 (0.62–1.01) | 0.055 |
| **Infant mortality (0-11 months of life)** |  |  |  |  |
| No IFA supplementation | 3879 | 167 | 1.00 (reference) |  |
| Any IFA supplementation | 3879 | 133 | 0.81 (0.66–1.00) | 0.051 |
| **Under five mortality (0-59 months of life)** |  |  |  |  |
| No IFA supplementation | 3879 | 223 | 1.00 (reference) |  |
| Any IFA supplementation | 3879 | 180 | 0.80 (0.65–0.98) | 0.037 |

60 missing values were excluded from the analysis.

^†^ Adjusted for province, pooled household wealth index, average coverage of BCG vaccination against tuberculosis (for mortality in neonatal and infant period), average coverage of measles vaccination (for under-five mortality), paternal level of attained education, paternal working status, maternal desire for pregnancy, sex of the child, birth rank and birth interval, maternal perception of birth size, timing of initiation of breastfeeding, number of antenatal care visits, place of delivery, delivery assistance, mode of delivery and year of birth.

BCG: Bacillus Calmette-Guerin; CI: Confidence interval. IFA: Iron-folic acid. HR: Hazard ratio.

**Figure S1: Effect of Maternal Antenatal Iron-Folic Acid Supplementation on Childhood Mortality Indicators of Most Recent Live Births 5 Years prior to Interview in Pakistan 2002–2012: Results of Multivariate Cox Proportional Hazard Regression Analyses Adjusted for Sampling Weight**

76 missing values were excluded from the analysis.

**^π^** Weighting was applied to compensate for the multistage cluster sampling design.

^†^ Adjusted for province, pooled household wealth index, average coverage of BCG vaccination against tuberculosis (for mortality in neonatal and infant period), average coverage of measles vaccination (for under-five mortality), paternal level of attained education, paternal working status, maternal desire for pregnancy, sex of the child, birth rank and birth interval, maternal perception of birth size, timing of initiation of breastfeeding, number of antenatal care visits, place of delivery, delivery assistance, mode of delivery and year of birth.

BCG: Bacillus Calmette-Guerin; CI: Confidence interval. IFA: Iron-folic acid. HR: Hazard ratio.

**Figure S2: Effect of Maternal Antenatal Iron-Folic Acid (IFA) Supplementation, Timing of Imitation of IFA Supplementation and Combined IFA with Other Antenatal Care Services Utility on Perceived Birth Size of Most Recent Live Births 5 Years prior to Interview in Pakistan 2002–2012: Results of Multivariate Poisson Regression Analyses**

52 missing values were excluded from the analysis of IFA supplementation, 521 missing values were excluded from the timing of initiation of IFA supplementation analysis and 76 missing values were excluded from the combined IFA supplementation and other ANC services used analysis.

^†^Adjusted for province of residence, pooled household wealth index, paternal level of attained education, paternal working status, maternal desire for pregnancy, sex of the child, birth rank and birth interval, number of antenatal care visits and year of birth.

ANC: antenatal care; CI: Confidence interval; IFA: Iron-folic acid; RR: Risk ratio.
